# Effects of Music on Pain and Anxiety During Otolaryngology Surgery: A Systematic Review and Meta‐analysis

**DOI:** 10.1002/oto2.70041

**Published:** 2024-11-19

**Authors:** Kenny Do, Eric Kawana, Kurtis Young, Harry H. Ching, Jo‐Lawrence Bigcas

**Affiliations:** ^1^ Department of Otolaryngology–Head & Neck Surgery Kirk Kerkorian School of Medicine at UNLV Las Vegas Nevada USA

**Keywords:** anxiety, music therapy, otolaryngology, pain, surgery

## Abstract

**Objective:**

Patients undergoing surgery commonly experience anxiety during their procedure which can negatively affect surgical outcomes. Music therapy has emerged as a promising noninvasive approach to reducing anxiety particularly in patients undergoing otolaryngology procedures.

The objective of this study is to evaluate the effectiveness of music therapy on anxiety and pain during these procedures.

**Data Sources:**

PubMed and Embase.

**Review Methods:**

A systematic search was conducted using the PRISMA approach to retrieve articles published between 1980 and March 2024. The search terms were applied to PubMed and Embase databases. The search term yielded a total of 669 articles on PubMed and 1027 articles on Embase, 5 of which met the inclusion criteria.

**Results:**

Five studies consisting of 4 randomized controlled trials and one case‐control studies were included in our study, resulting in a total of 381 patients undergoing various otolaryngology procedures. Music therapy was found to reduce preoperative, perioperative, and postoperative anxiety and pain levels compared to control groups. Furthermore, patients who received music therapy experienced improvements in physiological parameters such as heart rate and blood pressure, indicating a positive impact on stress.

**Conclusion:**

Music therapy offers a potentially cost‐effective and noninvasive method of reducing anxiety and pain in otolaryngological surgery patients. Our study indicates that music therapy can serve as a valuable addition to traditional pharmacological approaches in managing surgery‐related anxiety and pain. However, more research is needed to standardize music therapy protocols and compare its effect in comparison to other nonpharmacologic and pharmacologic modalities to optimize care for patients.

Patients undergoing surgery often experience feelings of anxiety, which may impact surgical outcomes both before and after the procedure.^1^ Several factors influencing surgical planning can influence aspects of anxiety experienced by patients, including surgical history, apprehension about potential outcomes, interactions with providers, home social support, and additional variables depending on the patient's situation. Research findings indicate that patients frequently express apprehension concerning the expected outcomes of a procedure.^1^, ^2^ Methods to reduce anxiety in patients include the use of medications, including benzodiazepines.^3^ However, patients might encounter adverse reactions, including delirium, dizziness, vomiting, nausea, dry mouth, and other adverse effects.^3^, ^4^ In recent years, new approaches have been developed to reduce anxiety in patients undergoing surgery, some of which include the use of aromatherapy, acupuncture, hypnosis, television, virtual reality, augmented reality, and even music therapy.^4^, ^5^, ^6^, ^7^, ^8^ These alternative treatments are inexpensive, non‐intrusive, and have minimal side effects, proving effective in easing anxiety and pain for patients undergoing surgery.^9^, ^10^ Furthermore, worries about addiction or adverse effects to certain medications can be alleviated with these alternative methods.^9^, ^10^

Patients undergoing a range of otolaryngology surgeries, including tonsillectomies, septoplasty, rhinoplasty, sinus surgery, cochlear implantation, thyroidectomy, and head and neck procedures, may encounter anxiety and pain both preoperatively and postoperatively. Various studies have shown that preoperative anxiety is correlated with increased pain following surgery and is associated with the increased need for analgesia as well.^11^ Music therapy has been shown to decrease anxiety and pain during various procedures, including total knee replacement, colonoscopies, coronary artery bypass grafting, and more.^12^, ^13^, ^14^ The core mechanism of music therapy involves diminishing the activation of the pituitary‐adrenal stress pathway, which leads to the reduction of cortisol release.^15^ Additionally, music therapy has the capability to soothe the autonomic nervous system, leading to decreased emotional responses, heart rate, and blood pressure.^15^ Various factors, such as the type of music, tempo, and duration of listening time have been shown to affect the level of anxiety and pain experienced by surgical patients.^16^

In accordance with the PICO guidelines, this systematic review addresses the following question: in adult patients who undergo various otolaryngology procedures or surgeries: Does listening to music relieve perioperative anxiety and pain compared to those who do not listen to music perioperatively? The hypothesis is that patients who are exposed to music perioperatively will have reduced anxiety and pain scores compared to patients in the control group. Secondary outcomes, such as systolic blood pressure, heart rate, and more, may be lower in the treatment group as well.

## Methods

### Study Design

**Figure 1:**
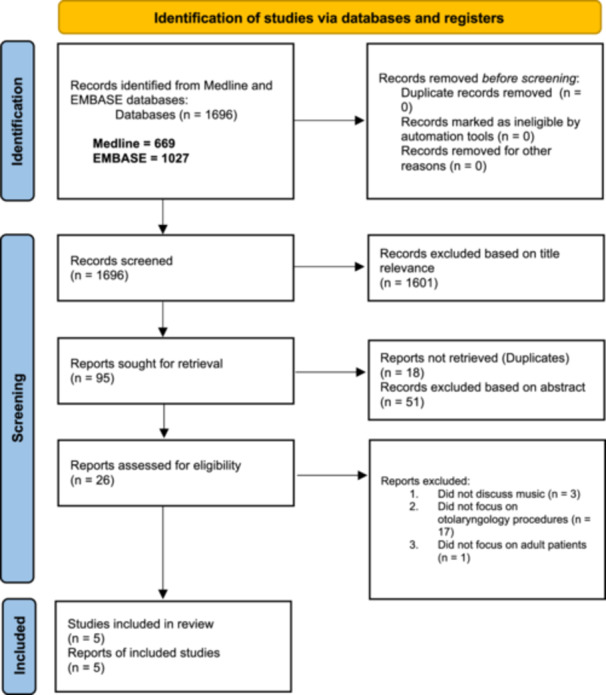
Study flow chart.^17^

This systematic review was reported according to the 2020 PRISMA guidelines.^17^ The PubMed and Embase databases were employed to retrieve articles for this study. Two independent investigators (KD and EK) used the search phrase, “music anxiety surgery,” in these 2 databases. To supplement, additional search terms in Appendix 1 were also queried in PubMed with several Boolean operators, including “AND” and “OR.” In total, 669 articles were produced on PubMed (years ranging from 1980 to March 2024). Although these search terms are extensive, there is a possibility that more studies may have been discovered if other search words or phrases were utilized. The full search terms in Appendix 1 were also inputted in Embase. This produced 1027 articles on Embase (years ranging from 1980 to March 2024). Figure 1 displays the systematic screening process involved in filtering these articles for their titles, abstracts, and full‐length papers. Articles were included if they investigated the effectiveness of music in reducing anxiety and pain for adult patients undergoing otolaryngology procedures and surgeries. Articles were not included in this study if they focused on surgeries or procedures that were not within the field of otolaryngology. They were also excluded if they did not discuss the use of music therapy for adult patients. The included studies and their characteristics are presented in Table 1.

**Table 1:** Anxiety and Pain Scores of Music Therapy Versus No Music Therapy

Authors	Type of study	Type of procedures	Type of anesthesia	Music administration	Number of patients in treatment	Number of patients in control	Pain Scores Music Group	Pain Scores Control Group	Preoperative Anxiety Score Music Group	Preoperative Anxiety Score Control Group	Postoperative Anxiety Score Music Group	Postoperative Anxiety Score Control Group	Risk of Bias Quality
Gogoularadja et al^18^	Randomized Control Study	Septoplasty	General Anesthesia	Patients selected calming music 1 day before surgery. Two 30‐minute sessions via headphones were conducted daily before surgery and for 2 days after surgery.	29	30	3.03 ± 1.35	6.67 ± 1.40	3.14 ± 1.71	6.73 ± 2.32	2.86 ± 2.33	8.93 ± 2.20	Good
Gökcek et al^19^	Randomized Double‐Blinded Study	Septorhinoplasty	General Anesthesia	Patients in the music group used headphones to hear relaxing music (pop, jazz, classical) at 65 dB via MP3 during OR preparation until general anesthesia was given	60	60	2.73 ± 1.28	3.61 ± 1.40	na	na	na	na	Good
Ortega et al^16^	Randomized Control Study	Nasal Bone Fracture Reduction	Local anesthesia with 2% lidocaine and 1:100,000 adrenaline was used via bilateral endo‐nasal block.	Patients listened to a 60‐80 BPM Spotify playlist through adjustable Bluetooth headphones for 10 minutes before, during, and after the procedure	17	19	3 ± 1.04	6 ± 1.79	27 ± 6.99	26 ± 5.52	23 ± 4.85	32 ± 4.60	Good
Casale et al^20^	Case Controlled Study	Video‐assisted endoscopic radiofrequency inferior turbinate volume reduction	Local anesthesia with mepivacaine and adrenaline	Patients chose favorite music (pop, jazz, classical, rock, or no preference). Music (50‐60 dB) played via stereo during RFVTR on one nostril; other nostril treated without music. Time break between each nostril was at least 5 minutes.	23	23	5.7 ± 2.42	6.7 ± 1.97	na	na	na	na	Good
Gomez‐Urquiza et al^21^	Randomized Controlled Clinical Trial	myringoplasty, stapedectomy, septoplasty, etc.	Did not mention	Patients in the waiting area, where they waited for 45 to 120 minutes, were entertained with relaxing music and scenic photographs until they were brought into the operating room.	60	60	na	na	30.83 ± 13.29	32.17 ± 13.29	na	na	Fair

### Study Selection

KD and EK screened articles for this systematic review and a senior researcher (KY) resolved conflicts that arose during the process. KD and EK first evaluated the relevance of 1696 article titles to the project's scope. Titles mentioning music and surgery during the perioperative period advanced to the second stage. After removing duplicates, KD and EK assessed the abstracts. Finally, they examined the full‐length papers, including only those discussing music in otolaryngology procedures in the final review. Articles that focused on alternative nonpharmacological interventions like virtual reality, those addressing procedures outside the realm of otolaryngology, and those not written in English were excluded from the study.

### Quality Assessment and Data Abstraction

**Figure 2:**
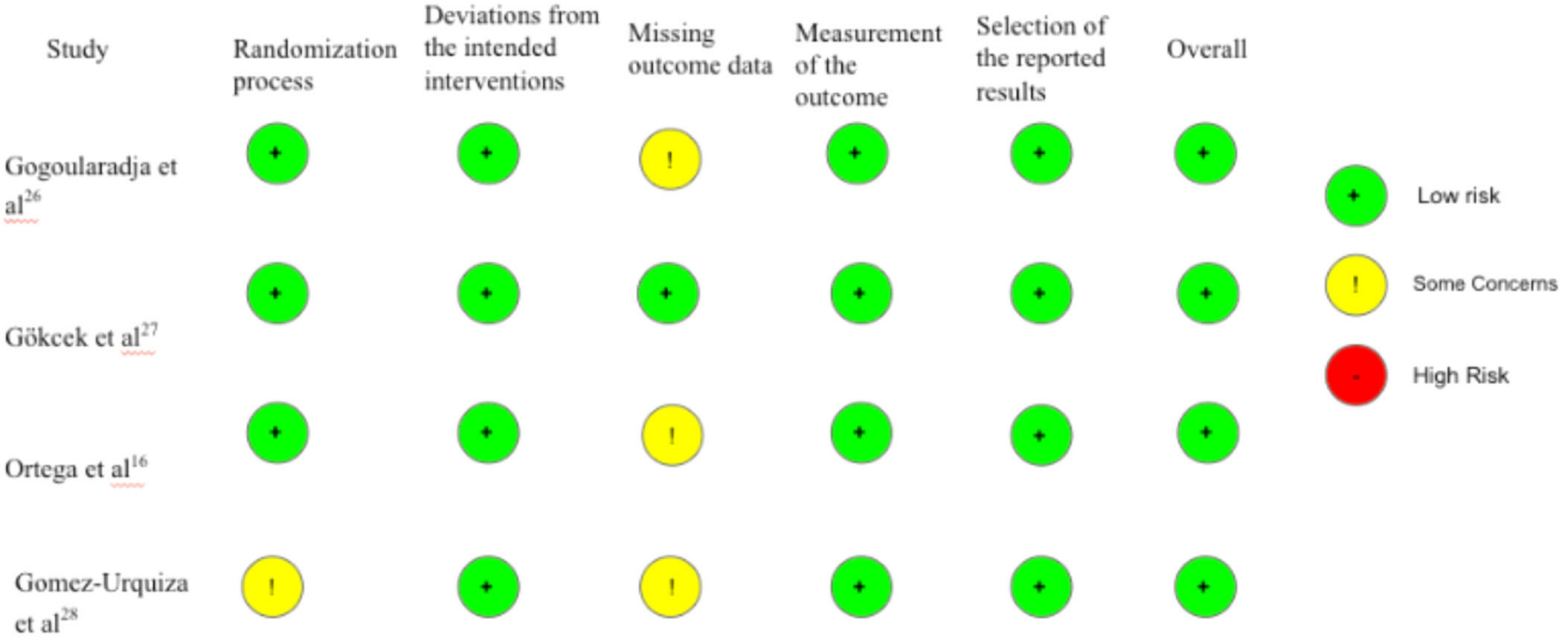
Cochrane risk of bias tool for randomized control trials assessment.^23^

Questionnaires provided by the National Institute of Health (NIH) Study Quality Assessment tool^22^ (Table 2) and the Cochrane Risk of Bias Tool^23^ (Figure 2) were used by KD and EK to assess the risk of bias of the 5 individual articles included in this systematic review. The NIH Risk of Bias Assessment for Case‐Control Studies^22^ consisted of 12 questions, where scores ranging from 9 to 12 was considered good quality, 6 to 8 was considered fair quality, and 0 to 5 was considered poor quality. The Cochrane Risk of Bias tool^23^ evaluated the randomized control trials as low, medium, or high bias across 6 different criteria.

**Table 2:** Risk of Bias Assessment of Case‐Control Studies

	Casale et al
Q1. Was the research question or objective in this paper clearly stated and appropriate?	Y
Q2. Was the study population clearly specified and defined?	Y
Q3. Did the authors include a sample size justification?	N
Q4. Were controls selected or recruited from the same or similar population that gave rise to the cases (including the same timeframe)?	Y
Q5. Were the definitions, inclusion and exclusion criteria, algorithms or processes used to identify or select cases and controls valid, reliable, and implemented consistently across all study participants?	Y
Q6.Were the cases clearly defined and differentiated from controls?	Y
Q7. If less than 100 percent of eligible cases and/or controls were selected for the study, were the cases and/or controls randomly selected from those eligible?	Y
Q8. Was there use of concurrent controls?	Y
Q9. Were the investigators able to confirm that the exposure/risk occurred prior to the development of the condition or event that defined a participant as a case?	Y
Q10. Were the measures of exposure/risk clearly defined, valid, reliable, and implemented consistently (including the same time period) across all study participants?	Y
Q11. Were the assessors of exposure/risk blinded to the case or control status of participants?	N
Q12. Were key potential confounding variables measured and adjusted statistically in the analyses? If matching was used, did the investigators account for matching during study analysis?	N
Final Quality Score	9
Rating	Good

### Data Analysis

For the meta‐analysis parts of the study, the SPSS software version 29 (IBM Corp.) was used for statistical analysis. For continuous variables that were common in the studies, the mean and standard deviation were calculated. A random effects model with restricted maximum likelihood (REML) estimation was used to calculate the mean differences of anxiety scores, VAS pain scores, and systolic blood pressure (SBD) of patients in the music and control groups.^24^, ^25^ For studies that only presented the median and interquartile ranges, Wan et al's equation was used to approximate the mean and standard deviations.^26^ Heterogeneity was evaluated using the *I* ^2^ index, with 0% to 50% being low, 50% to 75% moderate, and 75% to 100% being high.^27^ To address potential publication bias, SPSS software was used to create funnel plots for these individual studies, with an *α* value set at 0.05 for reference.^28^

## Results

After the systematic screening process detailed in Figure 1, 5 articles were included in the current study. The reasons for exclusion comprised studies that failed to evaluate the impact of music on otolaryngology patients, were not written in English, or were not full‐length peer‐reviewed publications. The articles that were included in this systematic review included 4 randomized control trials, and 1 case‐control study. Data concerning preoperative anxiety/pain scores and postoperative anxiety/pain scores were compiled from individual studies for this systematic review. Additional variables demonstrated in these articles, such as patient satisfaction scores, blood pressure, and more were examined as well. Table 1 displays the results from these individual studies.

### Study Characteristics

Across the 5 studies examining the effects of music on patients undergoing otolaryngology operations, there were 189 patients who underwent an ENT procedure and listened to music. For control, there were 192 patients who underwent an ENT procedure without listening to music. It is worth noting that in the study by Casale et al,^20^ the patients in the music intervention group also functioned as the control group. Music was administered to patients during surgery on 1 side, while no music was provided to the same patients through the contralateral side during endoscopic radiofrequency inferior turbinate reduction.^20^ The demographics of these patients can further be broken down into age and gender. For the music group, the average age was 37.4 years old while for the control group, the average age was 34.0 years old. The treatment group had 92 male patients and 75 female patients, and the control group had 89 male patients and 79 female patients. One study did not specify the gender of its participants.

Using the National Institute of Health (NIH) Study Quality Assessment tool^22^ (Table 2) and the Cochrane Risk of Bias Tool^23^ (Figure 2), the researchers evaluated the risk of bias of the 5 individual studies. After evaluation, it was determined that 4 of the studies in this systematic review were of good quality and 1 of the study was of fair quality.

### Qualitative Synthesis of Included Studies

Gogoularadja and Bakshi^18^ conducted a randomized control study that examined a total of 59 patients who underwent septoplasty under general anesthesia. Initially, 30 patients were in the music group and 30 patients were in the control group, but 1 patient in the music group was lost to follow‐up. In the treatment group, patients received headphone music just before the operation and 2 days following surgery, with each day having 2 30‐minute music sessions. Pain scores were lower in the music group compared to the control group on Day 0 (3.03 ± 1.35 vs 6.67 ± 1.40), Day 1 (1.83 ± 1.30 vs 6.28 ± 1.17), and Day 2 (0.79 ± 0.74 vs 4.67 ± 0.85). *P* < .001 between days and <.0001 between groups. Furthermore, the preoperative Generalized Anxiety Disorder‐7 (GAD‐7) score for the music group was 3.14 ± 1.71 compared to 6.73 ± 2.32 in the control group. The postoperative anxiety score for the music group was 2.86 ± 2.33 compared to 8.93 ± 2.20 in the control group. In this study, they demonstrated that both pain and anxiety was lower in patients who listened to perioperative music compared to those who did not listen to music.^18^

Another randomized control trial by Gökcek et al^19^ compared the VAS pain scores, patient satisfaction scores, and Ricker Sedation‐Agitation Scale scores in 60 patients who listened to music during the septorhinoplasty operation under general anesthesia and in 60 patients who did not listen to music during the surgery. The authors reported a statistically lower postoperative VAS pain scores for patients in the music group compared to the control group (2.73 ± 1.28 vs 3.61 ± 1.40 respectively). Moreover, 4 patients in the music group, as opposed to 9 patients in the control group, needed 0.5 mg/kg Pethidine HCL due to high levels of pain. Patient satisfaction score was statistically higher in the music group (73.30%) compared to the control patients (36.60%). The Ricker Sedation‐Agitation Scale scores showed that patients in the music group had improved transition from anesthesia than the control group, with the scores being 3.76 ± 1.64 and 5.11 ± 2.13 respectively. The author also reported that the music group had lower systolic arterial pressure (SAP), diastolic arterial pressure (DAP), and mean arterial pressure (MAP); however, these values were not statistically significant.^19^

Ortega et al studied 36 patients who underwent nasal fracture bone reduction surgery with local anesthesia, with 17 who listened to music and 19 who did not listen to music.^16^ The participants in the music group listened to music 10 minutes before the surgery, during the entire length of the surgery, and 10 minutes after the surgery. The preoperative State‐Trait Anxiety Inventory (STAI) score for patients in the music and control groups before the surgery was 27 ± 6.99 and 26 ± 5.52. However, after the operation, the music group had a lower anxiety score of 23 ± 4.85 compared to 32 ± 4.60 in the control group. Furthermore, the preoperative VAS pain scores were also similar between the 2 groups, but postoperatively, the VAS pain score was a 3 ± 1.04 in the music group and a 6 ± 1.79 in the control group. Although the systolic blood pressure of the music group did not change drastically, it was observed that their systolic blood pressure remained stable before, during, and after the surgery. In the control group, there was an increase in systolic blood pressure, peaking as high as 20 mmHg during the operation.^16^

Gomez‐Urquiza et al studied 180 participants undergoing various ENT operations, such as myringoplasty, stapedectomy, septoplasty, and more.^21^ There were 60 patients in the control group, 60 who observed scenic photographs, and 60 who observed photographs while listening to music. The researchers found that compared to the control group, patients in the music and photographic intervention groups had statistically significant lower heart rate, systolic blood pressure, and state anxiety. For preoperative state anxiety measured by the STAI scale, there was a mean difference of −9.41 between the music and control groups. There was also a mean difference of −2.78 for heart rate between the groups. Furthermore, compared to the only photographic group, the music intervention patients had statistically significant lower anxiety score (mean difference of −5.46) and SBP (mean difference of −2.26).^21^

Casale et al conducted a case‐control study that observed 23 patients with inferior turbinate hypertrophy undergoing radiofrequency inferior turbinate volume reduction with local anesthesia.^20^ During the procedure for 1 nostril, the 23 patients listened to music, while for the other nostril of the same patients, they did not listen to music. The VAS pain scores during the procedures were statistically lower in the music intervention (5.7 ± 2.42) than without the music intervention (6.7 ± 1.9). Moreover, systolic blood pressure during the procedure was found to be higher in the group without music (136.78 ± 16.8) compared to with music (133.5 ± 17.2).^2^, ^20^

### Meta‐analysis of Common Outcomes

**Figure 3:**
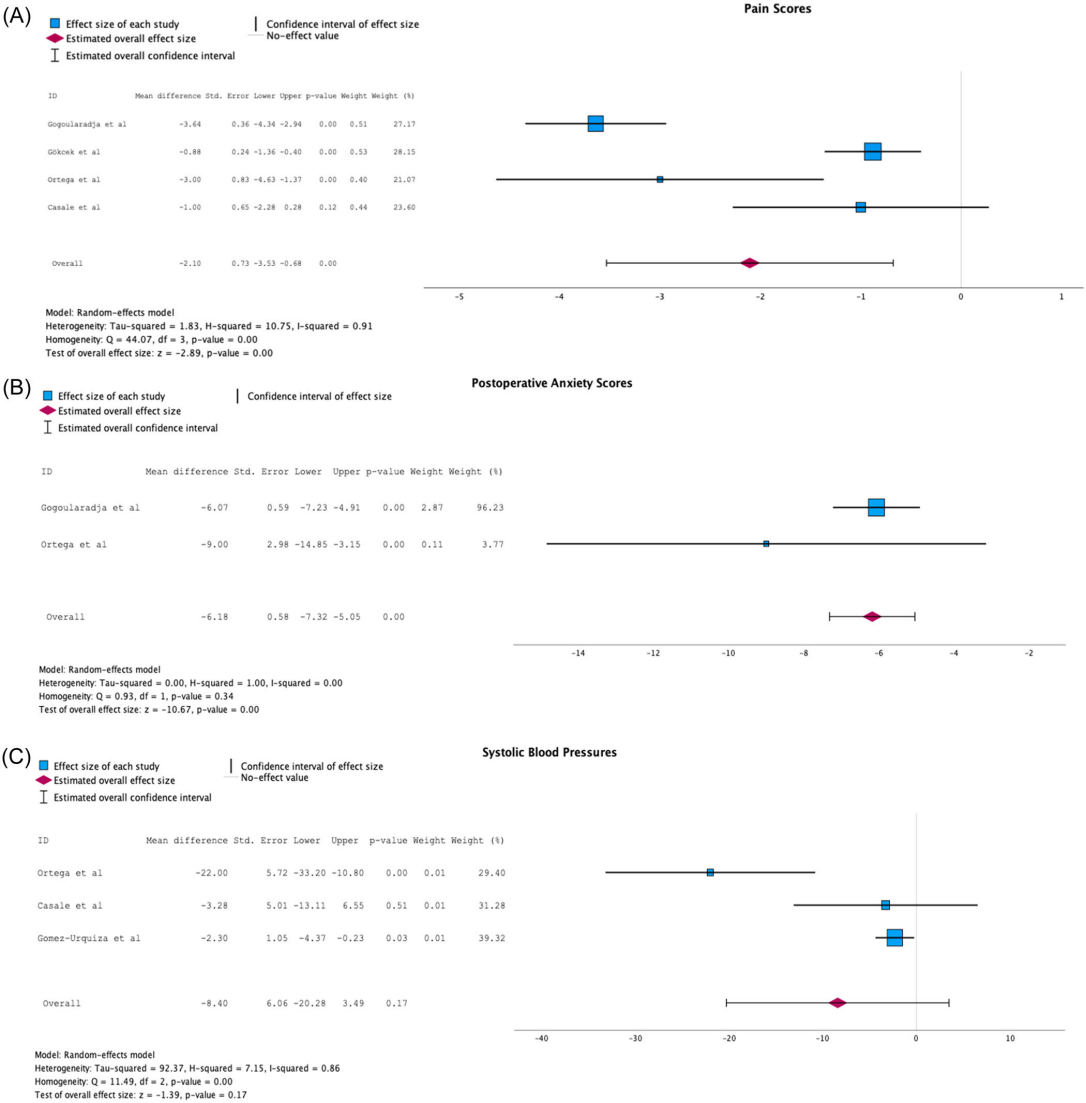
Forest plots of mean differences in (A). VAS pain scores, (B) Postoperative Anxiety Scores, and (C) Systolic blood pressures in treatment (music) and control group.

VAS pain scores were provided by 4 out of the 5 studies aggregated in this systematic review. The pooled VAS pain scores mean difference in Figure 3A was −2.10 points (95% CI: −3.53 to −0.68, *p*: .00). Two studies provided postoperative anxiety scores, and the pooled anxiety scores mean difference in Figure 3B was −6.18 points (95% CI: −7.32 to −5.05, *p*: .00). Three studies provided systolic blood pressure for patients in the music and control groups, and the pooled SBD mean difference in Figure 3C was −8.40 (95% CI: −20.28 to 3.49, *p*: .17). Heterogeneity was found to be high for the VAS pain score analysis (*I* ^2^ = 0.91), low for the postoperative anxiety scores analysis (*I* ^2^ = 0.00), and high for the systolic blood pressure analysis (*I* ^2^ = 0.86). The forest plots for these outcomes are displayed in Figure 3.

## Discussion

Approximately 310 million surgical operations are completed annually, and it is believed that up to 60% to 92% of patients experience preoperative anxiety before surgery.^29^, ^30^ Studies have shown that increased anxiety before and after surgeries can lead to poor wound healing, increased pain, and susceptibility to complications.^31^ A systematic review investigating the impact of perioperative anxiety on postoperative mortality in cardiac surgery revealed that the relative risk estimate for perioperative anxiety and late postoperative mortality was 1.81, nearly doubling the risk.^32^ Another study concluded that highly anxious patients before surgery reported high levels of pain following surgery and consumed more morphine than those who only had mild anxiety.^33^ Modalities to decrease anxiety and pain in surgery include pharmacologic and nonpharmacologic options. In the current systematic review, we examined the effects of music in reducing perioperative anxiety and pain in patients undergoing otolaryngology procedures.

The individual studies in this systematic review support the hypothesis that music intervention reduces anxiety for patients undergoing otolaryngology procedures. Gogoularadja and Bakshi,^18^ Ortega et al,^16^ and Gomez‐Urquiza et al^21^ compared anxiety levels between patients who were exposed to music or no music therapy. The scales used to assess anxiety in these patients included the Generalized Anxiety Disorder‐7 (GAD‐7) score and the State‐Trait Anxiety Inventory (STAI) score. The overall findings among these studies were that patients who received music therapy before and after surgery experienced less anxiety and stress than patients who were in the control group. Gogoularadja and Bakshi observed a 53.3% reduction in preoperative GAD‐7 anxiety score among patients who received music compared to those who did not before septoplasty.^18^ After the surgery, there was a 68.0% decrease in anxiety when comparing the control and music patients.^18^ In Ortega et al, both groups had similar preoperative STAI anxiety scores; however, there was a 28.3% decrease in STAI anxiety score between the control and music groups postsurgery.^16^ In Gomez‐Urquiza et al, there was a 4.2% decrease for preoperative STAI state anxiety, as the control group had a score of 32.17 and the music group had a score of 30.83.^21^

Postoperative pain reported by patients was lower for those who listened to music before and after the surgeries as well. Five out of the 6 included studies examined perioperative pain using a visual analogue scale (VAS). The decrease in pain scores in the treatment group compared to the control group was 54.6%, 24.4%, 50.0%, and 14.9% for Gogoularadja and Bakshi,^18^ Gökcek et al,^19^ Ortega et al,^16^ and Casale et al,^20^ respectively.

The perceived decrease in anxiety and pain for patients undergoing major surgeries after listening to music is not well understood. One possible mechanism is that music serves as a cognitive diversion for the brain, which in turn activates the cingulo‐frontal cortex and subsequently signaling the periaqueductal gray and the posterior thalamus to reduce pain.^34^, ^35^ Nonetheless, these individual studies support the idea that music therapy can serve as an simple and inexpensive modality to reduce pain and anxiety for patients undergoing otolaryngology operations. The studies included in this systematic review solely necessitated headphones, MP3 players, stereos, or digital music playlists. Through the utilization of these cost‐effective methods, healthcare providers can potentially decrease the quantity of medications that patients need. One systematic review reported that perioperative music decreases the requirement for opioid, midazolam, and even propofol use.^36^

Other fields have studied the use of music in reducing stress, anxiety, and pain following intervention. One randomized control trial reported that patients exposed to music before and during percutaneous renal biopsy had a score of 35.4 ± 6.2 anxiety and 5.0 ± 1.4 for pain. These numbers are lower than patients in the control group who had a 42.9 ± 9.0 for anxiety and 6.0 ± 0.9 for pain.^37^ Another systematic review assessing music group to control group in patients undergoing prostate biopsy reported decreased pain and anxiety as well.^38^ Keilani et al reported a preoperative anxiety score of 3.1 ± 2.3 and postoperative anxiety score of 1.21 ± 0.85 for patients undergoing tooth extraction surgery, both of which were statistically significant less than 6.12 ± 1.88 and 2.62 ± 1.30 for control patients respectively.^39^

Several factors may influence the role of music in each of the individual studies. First, the type of music that patients listen to is important in affecting how they respond to the operation. In 3 of the studies included in this systematic review, patients were given multiple music genres and were able to select their preferred songs.^18^, ^19^, ^20^ The authors of the other studies preselected slow, relaxing, and rhythmic music.^16^, ^21^, ^39^ One study examining the effectiveness of different music genres in alleviating anxiety for third molar extractions found that compared to soft rock and Turkish music, classical Western songs reduced the most anxiety score.^40^ Other studies suggested that slower and smooth instrumental sounds may be best for reducing anxiety.^41^

There are several limitations to address for this systematic review. First, various external factors such as the duration of music exposure, frequency of music sessions, method of music delivery (whether through headphones or speakers), room lighting, room temperature, type of anesthesia (local versus general), and other variables were not standardized across the 6 individual studies. Each of these variables has the potential to influence the actual impact of music intervention on perioperative anxiety and pain in otolaryngology. Furthermore, some of the studies did not use the same scale to assess anxiety. For example, the (GAD‐7) score and the State‐Trait Anxiety Inventory (STAI) score were used by different studies to report anxiety, which makes it difficult to synthesize an overall anxiety score for this systematic review. In the study by Gomez‐Urquiza et al, patients in the music group also received relaxing photographic sceneries of Granada. The presence of the photographs in this study may present as a confounding variable as to why patients in the music and photographic group experienced lower preoperative state anxiety score than patients in the control group.^21^

Future works may encompass comparing the effects of music on anxiety and pain for patients undergoing ENT procedures when compared to other nonpharmacologic modalities, such as virtual reality, augmented reality, and television. There is also potential value in comparing the effectiveness of music with pharmacologic options, such as opioids for pain and benzodiazepines for anxiety.

## Conclusion

Music therapy shows potential as a nonpharmacologic modality to alleviate anxiety and pain during otolaryngological surgery. By offering both a cost‐effective and non‐invasive method, music therapy offers an adjunctive or even completely alternative approach to pharmacologic management. Overall, this systematic review supports the potential for music therapy as an adjunctive therapy in the management of perioperative anxiety and pain with possible improvement in patient well‐being and surgical outcomes.

## Author Contributions

**Kenny Do**, study conceptualization, methodology development, systematic article screening, risk of bias assessment, data analysis, data interpretation, manuscript writing, manuscript revision; **Eric Kawana**, methodology development, systematic article screening, risk of bias assessment, data analysis, data interpretation, manuscript writing, manuscript revision; **Kurtis Young**, data analysis, data interpretation, manuscript revision; **Harry H. Ching**, data analysis, data interpretation, manuscript revision; **Jo‐Lawrence Bigcas**, study conceptualization, methodology development, methodology supervision, data analysis, data interpretation, manuscript revision.

## Disclosures

### Competing interests

None.

## Funding source

None.

## Data Availability

Data is public information and is available upon request.

## Appendix 1

Additional PubMed Search Terms:

(“music” or “music therapy” or “songs”) AND (“anxiety” or “pain” or “nervous” or “stress”) AND (“surgery” or “procedures”) AND (“otolaryngology” or “ENT” or “ears nose throat”).

Additional Embase Search Terms:

(‘music anxiety surgery’ OR ((‘music'/exp OR music) AND (‘anxiety’/exp OR anxiety) AND (‘surgery’/exp OR surgery)).
